# Preventive Effect of Epigallocatechin Gallate, the Main Component of Green Tea, on Acute Lung Injury Caused by Air Pollutants

**DOI:** 10.3390/biom12091196

**Published:** 2022-08-29

**Authors:** Ken-Ichiro Tanaka, Shunsuke Nakaguchi, Sachie Shiota, Yuka Nakada, Kaho Oyama, Okina Sakakibara, Mikako Shimoda, Akio Sugimoto, Masaki Ichitani, Takanobu Takihara, Hitoshi Kinugasa, Masahiro Kawahara

**Affiliations:** 1 Laboratory of Bio-Analytical Chemistry, Research Institute of Pharmaceutical Sciences, Musashino University, 1-1-20 Shinmachi, Nishitokyo 202-8585, Japan; 2 Central Research Institute, ITO EN, Ltd., 21 Mekami, Makinohara 421-0516, Japan

**Keywords:** air pollution, oxidative stress, epigallocatechin gallate, reactive oxygen species, neutrophil extracellular traps

## Abstract

Reducing the health hazards caused by air pollution is a global challenge and is included in the Sustainable Development Goals. Air pollutants, such as PM_2.5_, induce respiratory and cardiovascular disorders by causing various inflammatory responses via oxidative stress. Catechins and polyphenols, which are components of green tea, have various protective effects, owing to their antioxidant ability. The main catechin in green tea, epigallocatechin gallate (EGCG), is potentially effective against respiratory diseases, such as idiopathic pulmonary fibrosis and asthma, but its effectiveness against air-pollution-dependent lung injury has not yet been investigated. In this study, we examined the effect of EGCG on urban aerosol-induced acute lung injury in mice. Urban aerosol treatment caused increases in inflammatory cell counts, protein levels, and inflammatory cytokine expression in the lungs of ICR mice, but pretreatment with EGCG markedly suppressed these responses. Analyses of oxidative stress revealed that urban aerosol exposure enhanced reactive oxygen species (ROS) production and the formation of ROS-activated neutrophil extracellular traps (NETs) in the lungs of mice. However, ROS production and NETs formation were markedly suppressed by pretreating the mice with EGCG. Gallocatechin gallate (GCG), a heat-epimerized form of EGCG, also markedly suppressed urban aerosol-dependent inflammatory responses and ROS production in vivo and in vitro. These findings suggest that EGCG and GCG prevent acute lung injury caused by urban aerosols through their inhibitory effects on ROS production. Thus, we believe that foods and medications containing EGCG or GCG may be candidates to prevent the onset and progression of acute lung injury caused by air pollutants.

## 1. Introduction

Air pollution is a growing health hazard worldwide, and the number of deaths caused by air pollution (8.8 million per year) exceeds those caused by smoking [1]. Suspended particulate matter or fine particulate matter (PM_2.5_) contained in polluted air can remain in the air for a long time and easily reaches the periphery of the human lung to induce respiratory and cardiovascular diseases [1,2]. Because reducing the health hazards caused by air pollution is a global challenge, as stated in the Sustainable Development Goals [3], methods to prevent the onset and progression of respiratory and cardiovascular diseases caused by these air pollutants must be established as soon as possible.

In vitro and in vivo analyses have shown that oxidative stress plays a profound role in the onset and progression of health problems caused by air pollutants. Piao, C.H. et al. reported that the intranasal administration of PM_2.5_ exacerbated oxidative stress and inflammatory responses via the nuclear factor erythroid 2-related factor-2 signaling pathway in mice with ovalbumin-induced allergic rhinitis [4]. Additionally, PM_2.5_ was reported to promote inflammatory cell infiltration in the lungs of Sprague–Dawley rats by increasing the oxidative stress marker 8-epi-prostaglandin (PG)F_2α_ and decreasing the antioxidant protein superoxide dismutase [5]. In vitro studies using A549 cells (lung epithelial cells) have shown that PM_2.5_ increases the expression of intercellular adhesion molecule-1 by increasing oxidative stress and that the antioxidant N-acetylcysteine suppresses PM_2.5_-dependent inflammatory responses by inhibiting oxidative stress [6]. Furthermore, using an in vivo imaging system, we recently found that the production of reactive oxygen species (ROS) in the lungs of mice is enhanced by the administration of urban aerosols containing PM_2.5_ and that a thioredoxin derivative had a marked inhibitory effect on ROS production [7]. In a human epidemiologic study, Li, W. et al. found that the concentrations of PM_2.5_ and black carbon were positively correlated with myeloperoxidase (MPO), a marker of neutrophilic inflammation. The authors also found that the PM_2.5_ and sulfate concentrations were consistently positively correlated with urinary 8-epi-PGF_2α_ [8]. On the basis of these reports, compounds that can inhibit the oxidative stress caused by air pollutants may be candidates for reducing the health hazards of air pollution.

Green tea leaves are rich in catechins and other polyphenols. The catechins present in green tea are classified into two types: ester-type catechins, which have a gallate group attached to them, and free catechins, which do not have a gallate group. The major catechins are epicatechin, epigallocatechin, epicatechin gallate, and epigallocatechin gallate (EGCG), among which EGCG has the highest content in green tea. Catechins have been shown to exhibit various physiological activities, such as antioxidant, obesity prevention, antibacterial, and antiallergic effects [9,10]. Several reports have also examined the protective effect of EGCG on respiratory disease. For example, EGCG was found to exert an inhibitory effect on lipopolysaccharide- and H9N2 swine influenza virus-induced acute lung injury, and this effect was mediated by the suppression of Toll-like receptor 4 signaling activation [11,12]. In addition, green tea catechins were reported to ameliorate acute lung injury by inhibiting severe acute respiratory syndrome coronavirus replication and enhancing adaptive immunity and autophagy-dependent defense mechanisms [13]. However, no studies have analyzed the preventive effects of catechins, especially EGCG, against acute lung injury induced by air pollutants; accordingly, no studies have analyzed the details of their preventive mechanisms. Furthermore, green tea is sold as plastic bottled beverages and is often consumed in that form. EGCG, which is the major component of green tea, undergoes thermal epimerization during the tea-making sterilization processes. Thus, EGCG and its heat-epimerized form, GCG, are present in these plastic bottled beverages at roughly 35–40% EGCG and 45–50% GCG [14,15,16]. For example, commercial green tea beverages containing high doses of catechins have been shown to contain 960 mg/1200 mL green tea as total catechins, including EGCG and GCG (https://www.itoen-global.com/contents/en/oiocha/, accessed on 4 July 2022).

On the basis of the above-mentioned reports, we hypothesized that EGCG can exert a protective effect against air-pollution-dependent acute lung injury. Thus, in this study, we investigated the preventive effect of EGCG against urban aerosol-induced acute lung injury in mice. The mechanism by which EGCG prevents acute lung injury caused by urban aerosols was also examined, with a focus on oxidative stress and neutrophil extracellular traps (NETs). We also analyzed the protective effect of GCG against air-pollution-induced lung injury.

## 2. Materials and Methods

### 2.1. Chemicals and Animals

Diff-Quik staining solution was purchased from Sysmex (Kobe, Japan). Antibodies against citrullinated histone H3 (citrulline R2 + R8 + R17) (ab5103) and neutrophil elastase (ab21595) were purchased from Abcam (Cambridge, UK). An antibody against myeloperoxidase (22225-1-AP) was purchased from Proteintech Group, Inc. (Rosemont, IL, USA). Luminal-based chemiluminescent probe (L-012) and isoflurane were obtained from Fujifilm Wako Pure Chemical Corporation (Tokyo, Japan). A FastGene™ RNA Basic kit was obtained from Nippon Genetics Co., Ltd. (Tokyo, Japan), PrimeScript™ RT master mix (Perfect Real Time) was obtained from Takara Bio (Shiga, Japan), and THUNDERBIRD^®^ Next SYBR^®^ qPCR mix was obtained from Toyobo (Osaka, Japan). Novo-Heparin (5000 units) suitable for injection was purchased from Mochida Pharmaceutical (Tokyo, Japan). RAW264 cells were purchased from Riken BioResource Center (Tsukuba, Japan). ICR mice (6–7 weeks old, male) were purchased from Charles River (Yokohama, Japan). The experiments and procedures described herein were carried out in accordance with the Guide for the Care and Use of Laboratory Animals as adopted and promulgated by the National Institutes of Health and were approved by the Animal Care Committee of Musashino University.

### 2.2. Urban Aerosol Particle Suspension

Urban aerosol particulate matter was obtained from the National Institute for Environmental Studies (NIES; Tsukuba, Japan). These urban aerosol particles were collected by air filters placed in the central ventilation systems of buildings in central Beijing. An analysis of the urban aerosol particle composition was previously reported [17] and can be found on the NIES website (https://www.nies.go.jp/labo/crm-e/aerosol.html, accessed on 4 July 2022). For the animal studies, urban aerosol particles were suspended in sterile saline and administered to mice. For the cell experiments, the urban aerosol particles were suspended in ultrapure water, and the suspension was directly added to the medium.

### 2.3. Treatment of Mice with Urban Aerosols, EGCG and GCG

Mice were intraperitoneally administered EGCG (50–200 mg/kg) or GCG (100–200 mg/kg) in sterile saline 1 h before the intratracheal administration of the urban aerosol particle suspension. After anesthetization with isoflurane, the mice were intratracheally administered urban aerosols (0.2–2.0 mg/mouse) in sterile saline using a P200 micropipette. During administration, the nostrils of the mice were blocked so that the solution was inhaled from the mouth into the respiratory tract as the mouse breathed. The control group received sterile saline intraperitoneally and sterile saline intratracheally, the urban aerosol treatment group received sterile saline intraperitoneally and urban aerosol particle suspension in sterile saline intratracheally, and the EGCG (or GCG) group received EGCG (or GCG) intraperitoneally and urban aerosol particle suspension intratracheally.

### 2.4. Preparation of Bronchoalveolar Lavage Fluid (BALF) and Immunoblotting Analysis

BALF was collected by cannulating the trachea and lavaging the lung twice with 1 mL of sterile saline containing 50 units/mL heparin. Approximately 1.8 mL of BALF was recovered from each mouse, and the total cell number in the BALF was counted using a hemocytometer. After centrifugation in a Cytospin^®^4 instrument (Thermo Fisher Scientific, Waltham, MA, USA), the cells were stained with Diff-Quik reagents, and the ratio of neutrophils to the total cell number was determined. The amount of protein present in the BALF was evaluated by the Bradford method. The amount of double-stranded DNA (dsDNA) present in the BALF was evaluated using a NanoDrop^TM^ Lite (Thermo Fisher Scientific) a Quant-iT™ PicoGreen^®^ dsDNA Assay Kit (Thermo Fisher Scientific).

The BALF samples (10 µL) were then applied to a NuPAGE^®^ Novex 4–12% Bis-Tris gel (Thermo Fisher Scientific) and subjected to electrophoresis. The samples were loaded on NuPAGE Novex 4–12% Bis-Tris protein gels (Thermo Fisher Scientific) and electrophoresed at a constant voltage of 180 V, after which the proteins were transferred to polyvinylidene difluoride membranes using an iBlot^®^ 7-minute blotting system (Thermo Fisher Scientific). The membranes were blocked with 5% non-fat dry milk at room temperature for 1 h and incubated overnight with rabbit anti-citrullinated histone H3 antibody (1:1000 dilution), rabbit anti-neutrophil elastase antibody (1:1000 dilution), or rabbit anti-myeloperoxidase antibody (1:1000 dilution) in a solution of 5% bovine serum albumin, 1× Tris-buffered saline (TBS), and 0.1% Tween 20. The following day, the membranes were incubated for 1 h with horseradish peroxidase-linked goat anti-rabbit IgG antibody (1:2000 dilution) in 1× TBS containing 0.1% Tween 20. Finally, the protein bands were visualized using SuperSignal™ West Dura Extended Duration Substrate (Thermo Fisher Scientific). The band intensities were quantitated using ImageJ software (version 1.39u) (Bethesda, MD, USA).

### 2.5. In Vivo ROS Measurement by Imaging Analysis

In vivo ROS imaging in mice was performed as previously described [18,19,20] using a FUSION chemiluminescence imaging system (Vilber Lourmat, Collégien, France). The mice were intraperitoneally administered the ROS-sensing chemiluminescent probe L-012 in sterile saline (75 mg/kg) 24.5 h after the administration of the urban aerosol particle suspension. Fifteen minutes after L-012 administration, the mice were euthanized, and their lungs were promptly dissected and imaged (5-min exposure). All data were analyzed using FUSION chemiluminescence imaging system software.

### 2.6. Real-Time Reverse Transcription (RT) Polymerase Chain Reaction (PCR) Analysis

Total RNA was extracted from lung tissue or RAW264 cells using an RNeasy kit in accordance with the manufacturer’s protocol. The samples were reverse-transcribed using the PrimeScript RT master mix described above. The synthesized cDNA was used in the real-time PCR experiments with THUNDERBIRD Next SYBR qPCR mix and analyzed with a Bio-Rad (Hercules, CA, USA) CFX96™ real-time system and CFX Manager™ software. Specificity was confirmed by electrophoretic analysis of the reaction products and the inclusion of template- or reverse-transcriptase-free controls. To normalize the amount of total RNA present in each reaction, glyceraldehyde-3-phosphate dehydrogenase (*Gapdh*) cDNA was used as an internal standard. Primers were designed using the Primer-BLAST website (https://www.ncbi.nlm.nih.gov/tools/primer-blast/, accessed on 4 July 2022). Primer sequences are described in Supplementary Figure S1.

### 2.7. Cell Culture and In Vitro ROS Measurement

RAW264 cells (a mouse macrophage-like cell line) were cultured in Dulbecco’s Modified Eagle Medium supplemented with 10% fetal bovine serum. The cells were cultured in a humidified atmosphere of 95% air with 5% CO_2_ at 37 °C and precultured in black 96-well microplates (2 × 10^4^ cells/well). After incubation for 24 h, the cells were incubated with the ROS indicator 2’,7’-dichlorodihydrofluorescein diacetate (H_2_DCFDA, 10 µM) for 60 min. The cells were then treated with EGCG (2.5–10 μM) or GCG (2.5–10 μM) prior to the addition of urban aerosols to the medium. After 24 h, the ROS levels were measured using a microplate reader (Tecan, Kawasaki, Japan; excitation: 480 nm, emission: 530 nm).

### 2.8. Statistical Analysis

All data are expressed as the mean ± SEM. Significant differences among each group were examined using a one-way analysis of variance followed by Tukey’s multiple comparison. SPSS 24 software was used for all statistical analyses. Probability values of *p* < 0.05 were considered to indicate statistical significance.

## 3. Results

### 3.1. Mouse Model of Urban Aerosol-Induced Acute Lung Injury

**Figure 1:**
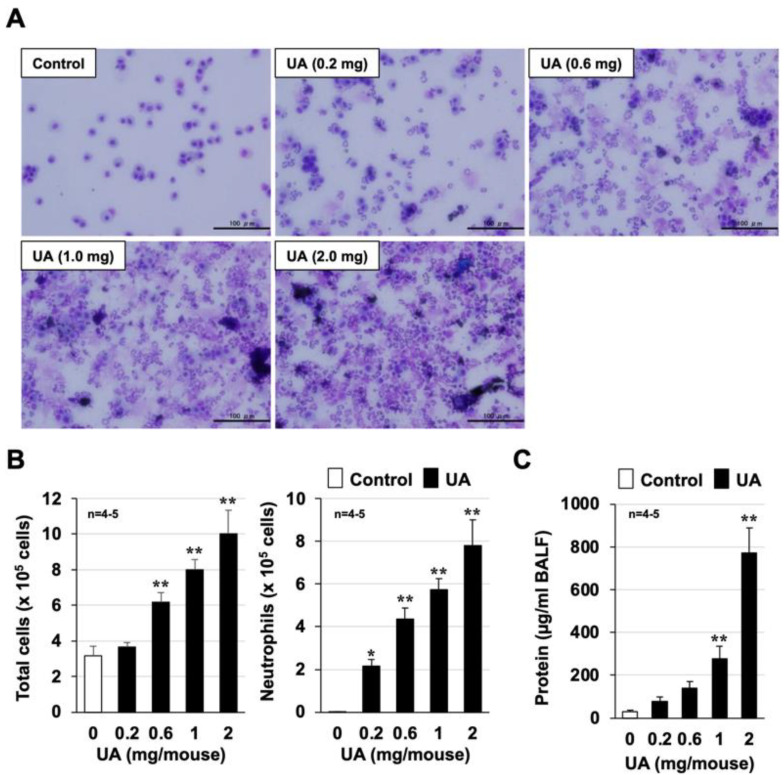
**Urban aerosol-induced lung injury.** Male ICR mice were intratracheally administered an urban aerosol particle suspension (0.2–2.0 mg/mouse) or sterile saline (Control). BALFs were prepared 24 h after the intratracheal administration. (**A**) BALF cells were deposited onto slides using a Cytospin 4 cytocentrifuge, then stained with Diff-Quik reagents and visualized under a light microscope (scale bar, 100 µm). (**B**) The numbers of total cells and neutrophils were determined. (**C**) The amount of protein present in the BALF was determined by the Bradford method. Values are expressed as the mean ± SEM; * *p* < 0.05; ** *p* < 0.01. (* vs. Control).

We first established a mouse model of urban aerosol-induced acute lung injury that improved on our previous report [7]. Figure 1A shows representative images from each group. The number of inflammatory cells in BALF was markedly increased 24 h after urban aerosol administration. The cell count results showed that the urban aerosols induced a dose-dependent increase in the total number of inflammatory cells in BALF. As shown in the right panel of Figure 1B, the majority of these increased inflammatory cells were neutrophils. The protein concentration in BALF is an indicator of lung injury and edema. As shown in Figure 1C, urban aerosol administration induced a dose-dependent increase in the amount of protein in BALF and in the number of inflammatory cells. These results demonstrate the successful establishment of a mouse model to assess the extent of air-pollution-dependent acute lung injury.

### 3.2. Effect of EGCG on Urban Aerosol-Induced Acute Lung Injury and Inflammatory Responses

**Figure 2:**
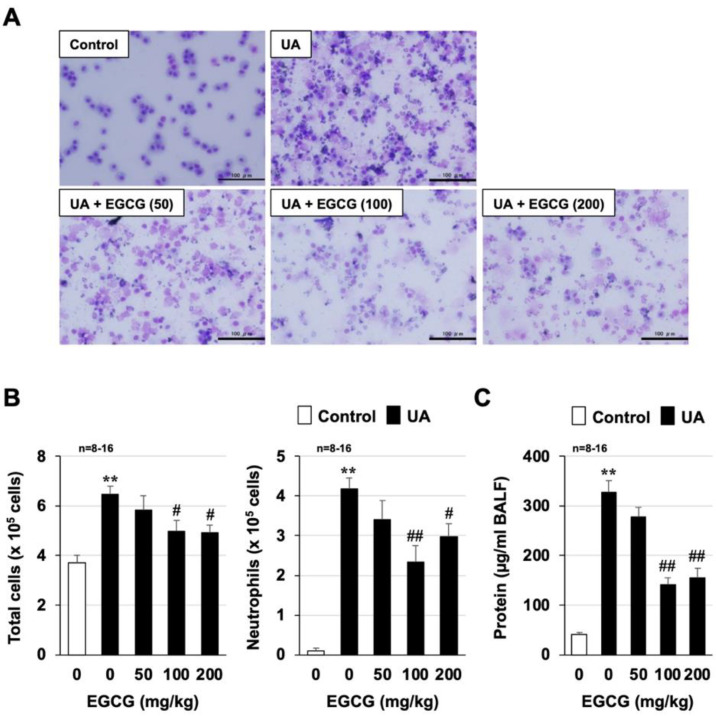
**Effect of EGCG on urban aerosol-induced lung injury**. Male ICR mice were intraperitoneally administered EGCG (50–200 mg/kg in sterile saline) or sterile saline 1 h before intratracheal administration of urban aerosol (UA) particle suspension (1.0 mg/mouse) or sterile saline (Control). BALFs were prepared 24 h after the intratracheal administration. (**A**) BALF cells were deposited onto slides using a Cytospin 4 cytocentrifuge, then stained with Diff-Quik reagents and visualized under a light microscope (scale bar, 100 µm). (**B**) The numbers of total cells and neutrophils were determined. (**C**) The amount of protein present in the BALF was determined by the Bradford method. Values are expressed as the mean ± SEM; ^#^ *p* < 0.05; ** or ^##^ *p* < 0.01. (^#^ vs. UA).

Next, using the established mouse model described above, we examined the effects of EGCG on acute lung injury caused by urban aerosol treatment. We used EGCG doses that had previously been reported to be effective in animal models of other inflammatory diseases [21,22]. As shown in Figure 2A,B, urban aerosol treatment (1 mg/mouse) increased the total number of leukocytes in BALF, especially neutrophils. In mice pretreated with EGCG (50–200 mg/kg), exposure to urban aerosols also induced an increase in the total number of leukocytes, especially neutrophils, but the increase was significantly less than in the urban aerosol group. EGCG administration dose-dependently inhibited the increase in the total number of leukocytes, with the most pronounced inhibition at 100 mg/kg EGCG. As shown in Figure 2C, the BALF protein concentrations were increased by urban aerosol treatment, and this increase was significantly inhibited in a dose-dependent manner by pretreatment with EGCG.

**Figure 3:**
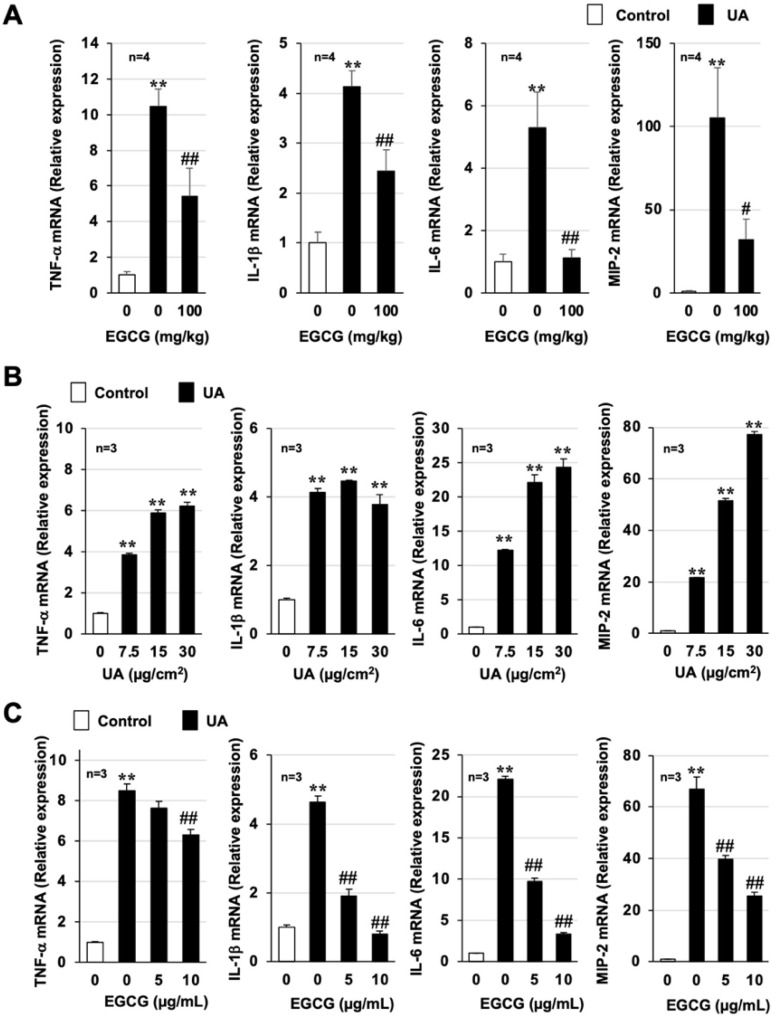
**Effect of EGCG on urban aerosol-dependent inflammatory responses**. (**A**) Male ICR mice were intraperitoneally administered EGCG (100 mg/kg in sterile saline) or sterile saline 1 h before intratracheal administration of urban aerosol particle suspension (1.0 mg/mouse) or sterile saline (Control). Total RNA was extracted from the lungs 24 h after urban aerosol particle suspension administration and subjected to real-time RT-PCR using a specific primer set for each gene. Values were normalized to *Gapdh* and are expressed relative to the control. (**B**,**C**) RAW264 cells were treated with urban aerosols (7.5–30 µg/cm^2^) (**B**) or EGCG (5–10 μg/mL) prior to the addition of urban aerosols (30 µg/cm^2^) (**C**) to the medium. Total RNA was extracted from the RAW264 cells 24 h after the addition of urban aerosols and subjected to real-time RT-PCR using a specific primer set for each gene. Values were normalized to *Gapdh* and are expressed relative to the control. Values are expressed as the mean ± SEM; ^#^ *p* < 0.05; ** or ^##^ *p* < 0.01. (^#^ vs. UA).

Air pollutants, such as PM_2.5_, promote inflammatory responses in the lungs and lung injury through their ability to induce the production of inflammatory cytokines [23]. Thus, proinflammatory cytokine expression was monitored using real-time RT-PCR. As shown in Figure 3A and Supplementary Figure S2, urban aerosol treatment significantly induced the mRNA expression of tumor necrosis factor-α (*Tnf-*α), interleukin-1β (*Il-1*β), *Il-6*, macrophage inflammatory protein 2 (*Mip2*), and keratinocyte-derived chemokine (*Kc*). However, the increased expression of these cytokines by urban aerosols was inhibited by pretreatment with 100 mg/kg EGCG. Next, we used RAW264 cells to determine whether EGCG directly inhibits urban aerosol-dependent induction of proinflammatory cytokines. Similar to the in vivo results, urban aerosol treatment induced the expression of proinflammatory cytokines in RAW264 cells in a dose-dependent manner (Figure 3B), and these inductions were significantly inhibited in a dose-dependent manner by pretreatment with EGCG (Figure 3C). Together, the data shown in Figure 2 and Figure 3 suggest that EGCG has an inhibitory effect on acute lung injury caused by urban aerosols by exerting anti-inflammatory effects.

### 3.3. Effect of EGCG on Urban Aerosol-Induced Oxidative Stress

**Figure 4:**
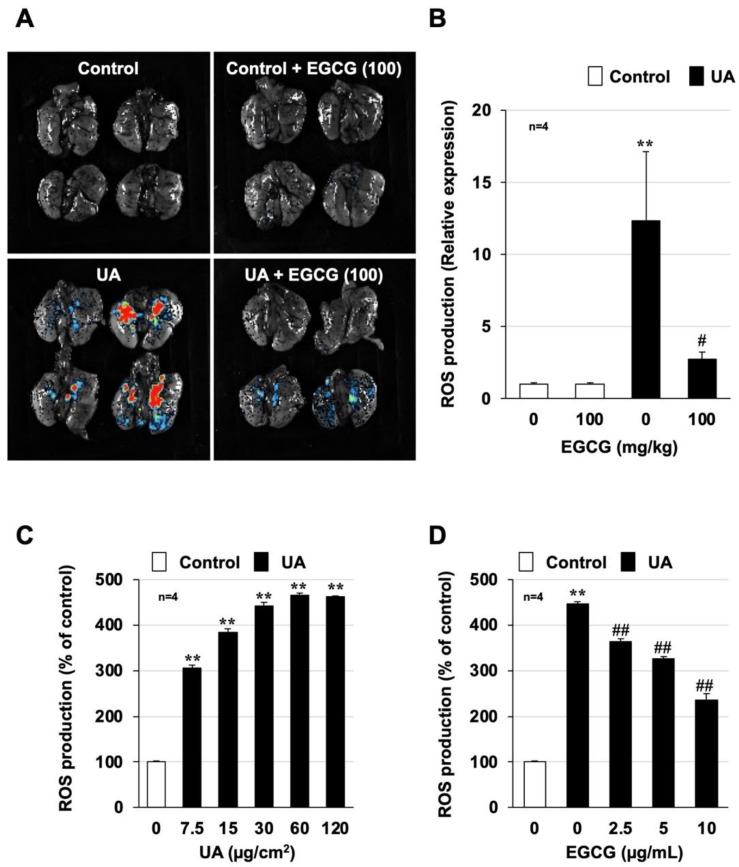
**Effect of EGCG on urban aerosol-induced ROS production**. (**A**,**B**) Male ICR mice were intraperitoneally administered EGCG (100 mg/kg in sterile saline) or sterile saline 1 h before intratracheal administration of urban aerosol particle suspension (1.0 mg/mouse) or sterile saline (Control). Luminescent probe (L-012, 75 mg/kg) was administered 24 h after administration of the urban aerosol particle suspension. (**A**) Isolated lungs were imaged using a FUSION chemiluminescence imaging system. (**B**) The summed pixel intensity of the ROS signal was determined using standard FUSION software. (**C**,**D**) RAW264 cells were treated with urban aerosols (7.5–120 µg/cm^2^) (**C**) or EGCG (2.5–10 μg/mL) prior to the addition of urban aerosols (30 µg/cm^2^) (**D**) to the medium. The cells were precultured with H_2_DCFDA (10 µM) for 60 min prior to EGCG and urban aerosol treatment. After 24 h, the ROS levels were measured using a microplate reader. Values represent the mean ± SEM; ^#^ *p* < 0.05; ** or ^##^ *p* < 0.01. (^#^ vs. UA).

ROS play an important role in the development and progression of various lung disorders, such as acute respiratory distress syndrome (ARDS) and idiopathic pulmonary fibrosis [24,25]. Oxidative stress is also deeply involved in lung injury and inflammatory responses induced by air pollutants [26,27]. Thus, the increase in ROS production due to urban aerosol administration was monitored using a FUSION in vivo imaging system. As shown in Figure 4A, an increase in the area that was stained red was observed in mouse lungs after urban aerosol administration, indicating increased ROS production. However, EGCG pretreatment (100 mg/kg) resulted in very little red staining after urban aerosol treatment, indicating that the ROS production was markedly inhibited. Quantitative analysis showed that 12.3 times more ROS were induced in the urban aerosol-treated group and 2.7 times more ROS were induced in the EGCG-pretreated group than in the control group, indicating that EGCG significantly suppressed ROS production (Figure 4B). We next analyzed urban aerosol-induced ROS production using an in vitro system similar to that used to investigate cytokine production. Treatment of RAW264 cells with urban aerosols (7.5–120 µg/cm^2^) induced ROS production in a dose-dependent manner in the in vitro experimental system (Figure 4C). Pretreatment with EGCG (2.5–10 µg/mL) significantly suppressed urban aerosol-dependent ROS production in RAW264 cells (Figure 4D).

In addition, we performed in vitro analysis using inflammatory cells recovered from BALF. As shown in Supplementary Figure S3, EGCG (2.5–10 µg/mL) significantly suppressed ROS production in inflammatory cells recovered from the BALF of urban aerosol-treated mice. Furthermore, the inhibitory effect of EGCG on urban aerosol-dependent ROS production was equal to or greater than that of the antioxidant N-acetylcysteine under our experimental conditions (Supplementary Figure S4). These results suggest that EGCG attenuates acute lung injury and inflammatory responses caused by urban aerosols by suppressing oxidative stress.

### 3.4. Effect of EGCG on Urban Aerosol-Induced NET Formation

**Figure 5:**
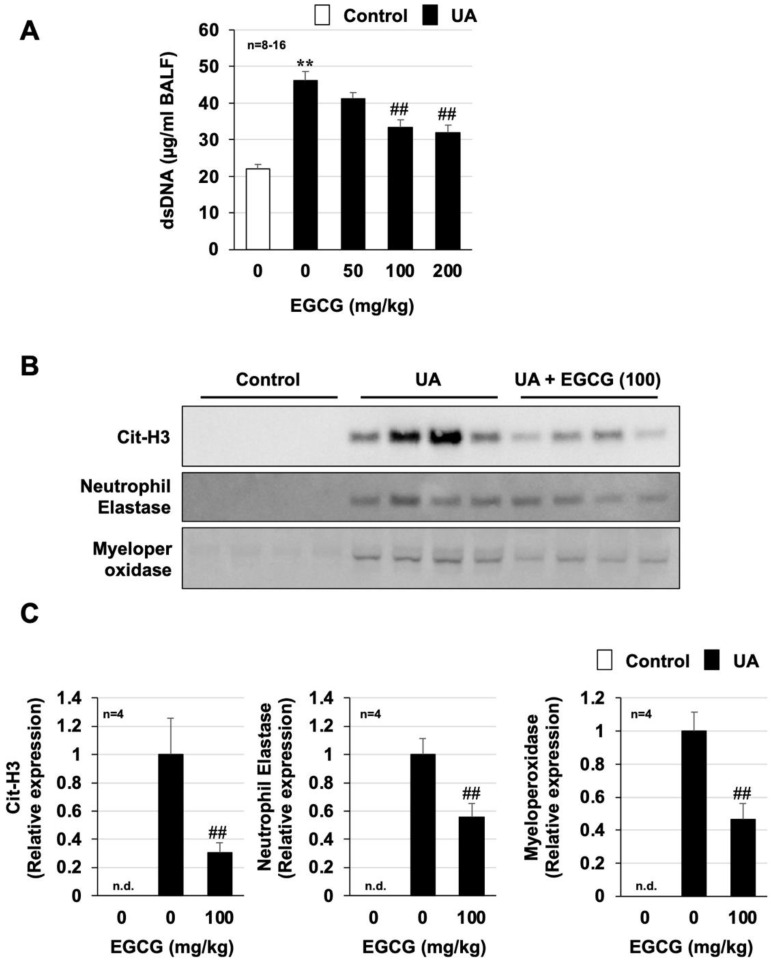
**Effect of EGCG on urban aerosol-induced neutrophil extracellular trap formation**. Male ICR mice were intraperitoneally administered EGCG (50–200 mg/kg in sterile saline) or sterile saline 1 h before intratracheal administration of urban aerosol particle suspension (1.0 mg/mouse) or sterile saline (Control). BALFs were prepared 24 h after the intratracheal administration. (**A**) The amount of double-stranded DNA (dsDNA) present in the BALF was determined using a NanoDrop Lite. (**B**) BALF samples (10 µL) were analyzed by immunoblotting with antibodies against Cit-H3, neutrophil elastase, and myeloperoxidase. (**C**) The intensities of the bands were determined using ImageJ software. n.d. indicates not detected. Values are expressed as the mean ± SEM; ** or ^##^ *p* < 0.01 (^#^ vs. UA).

NETs are composed of decondensed chromatin fibers and cytoplasmic protein, such as neutrophil elastase and MPO [28]. The citrullination of histones H3 and H4 is essential for chromatin dissociation and requires ROS-dependent activation of peptidylarginine deiminase 4 [29]. Thus, we analyzed the amount of dsDNA in BALF as a key indicator of NET formation. As shown in Figure 5A, the amount of dsDNA in BALF, which was elevated by intratracheal administration of urban aerosols, was dose-dependently suppressed by the preadministration of EGCG. The inhibitory effect of EGCG on urban aerosols-dependent dsDNA increase was also confirmed by another method using Quant-iT™ PicoGreen^®^ dsDNA assay kits (Supplementary Figure S5). Next, the expression levels of other NET indicators (citrullinated histone H3 (Cit-H3), neutrophil elastase, and MPO) were analyzed by Western blotting. As shown in Figure 5B,C, the expression of these NET indicators was significantly increased by urban aerosol administration but was undetectable in the control group. The EGCG-pretreated group showed significantly reduced urban aerosol-dependent increases in these NET indicators. On the basis of these results, the preadministration of EGCG appeared to prevent urban aerosol-induced lung injury by inhibiting ROS-dependent activation of NET formation.

### 3.5. Effect of GCG on Urban Aerosol-Induced Acute Lung Injury and Inflammatory Responses

**Figure 6:**
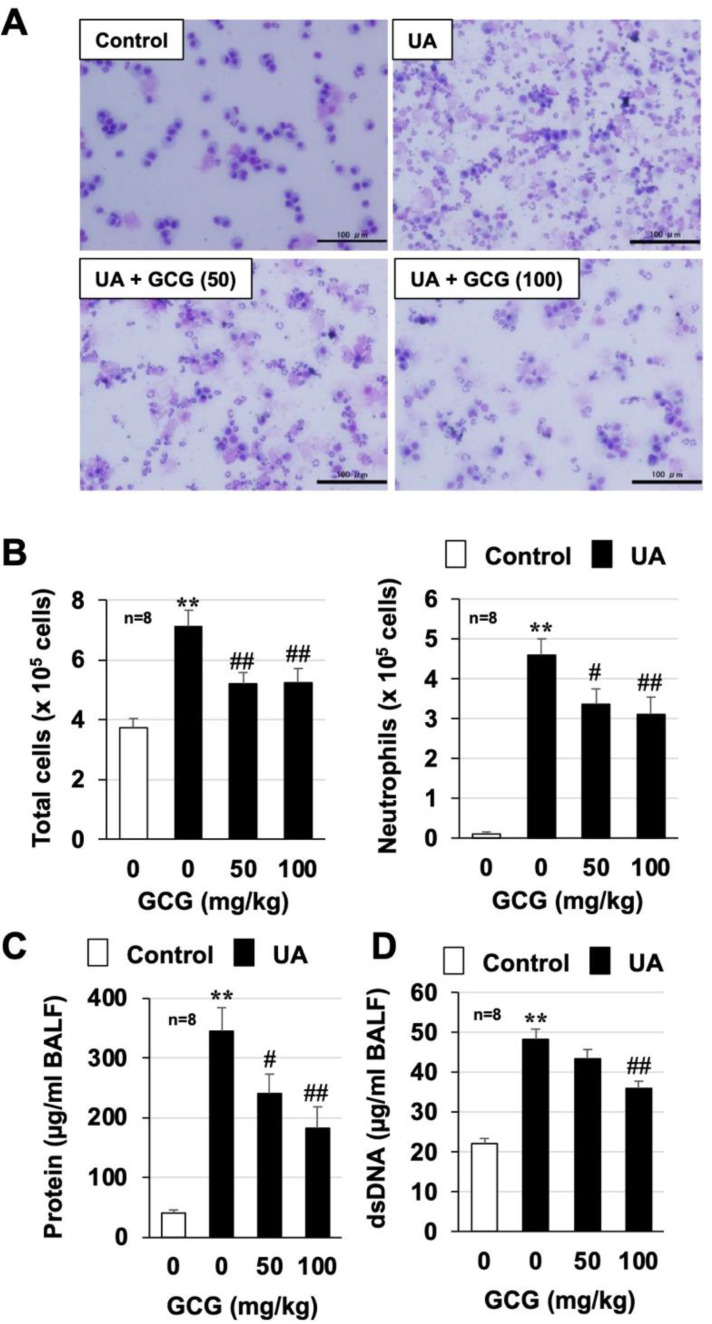
**Effect of GCG on urban aerosol-induced lung injury**. Male ICR mice were intraperitoneally administered GCG (50, 100 mg/kg in sterile saline) or sterile saline 1 h before intratracheal administration of urban aerosol particle suspension (1.0 mg/mouse) or sterile saline (Control). BALFs were prepared 24 h after the intratracheal administration. (**A**) BALF cells were deposited onto slides using a Cytospin 4 cytocentrifuge, then stained with Diff-Quik reagents and visualized under a light microscope (scale bar, 100 µm). (**B**) The numbers of total cells and neutrophils were determined. (**C**) The amount of protein present in the BALF was determined by the Bradford method. (**D**) The amount of double-stranded DNA (dsDNA) present in the BALF was determined using a NanoDrop Lite. Values are expressed as the mean ± SEM; ^#^ *p* < 0.05; ** or ^##^ *p* < 0.01. (^#^ vs. UA).

**Figure 7:**
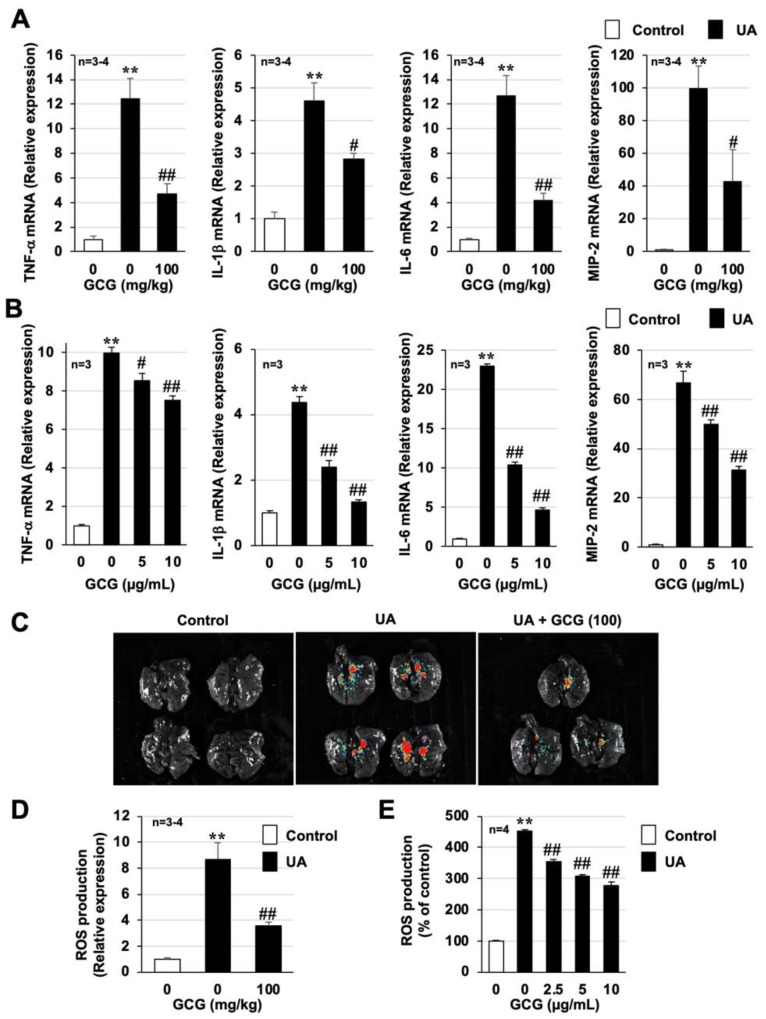
**Effect of GCG on urban aerosol-induced ROS production and inflammatory responses**. (**A**,**C**,**D**) Male ICR mice were intraperitoneally administered GCG (100 mg/kg in sterile saline) or sterile saline 1 h before intratracheal administration of urban aerosol particle suspension (1.0 mg/mouse) or sterile saline (Control). (**A**) Total RNA was extracted from the lungs 24 h after administration of urban aerosol particle suspension and subjected to real-time RT-PCR using a specific primer set for each gene. Values were normalized to *Gapdh* and are expressed relative to the Control. (**C**) Luminescent probe (L-012, 75 mg/kg) was administered 24 h after administration of the urban aerosol particle suspension. Isolated lungs were imaged using a FUSION chemiluminescence imaging system. (**D**) The summed pixel intensity of the ROS signal was determined using standard FUSION software. (**B**,**E**) RAW264 cells were treated with GCG (5–10 or 2.5–10 μg/mL) prior to the addition of urban aerosols (30 µg/cm^2^) to the medium. (**B**) Total RNA was extracted from the RAW264 cells 24 h after the addition of urban aerosols and subjected to real-time RT-PCR using a specific primer set for each gene. Values were normalized to *Gapdh* and are expressed relative to the Control. (**E**) The cells were precultured with H_2_DCFDA (10 µM) for 60 min prior to GCG and urban aerosol treatment. After 24 h, the ROS levels were measured using a microplate reader. Values represent the mean ± SEM; ^#^ *p* < 0.05; ** or ^##^ *p* < 0.01. (^#^ vs. UA).

As described in the Introduction, EGCG and its heat-epimerized form, GCG, are present in plastic bottled beverages [14,15]. Therefore, we analyzed the protective effect of GCG against air-pollution-induced lung injury. As shown in Figure 6 and Supplementary Figure S5, urban aerosol treatment increased total inflammatory cell counts, neutrophil counts, and protein and dsDNA levels in BALF, but GCG pretreatment significantly suppressed these increases. The dose at which an inhibitory effect was observed was similar to that of EGCG. GCG pretreatment also showed a significant inhibitory effect on the urban aerosol-induced increased expression of inflammatory cytokines in lung tissue and RAW264 cells (Figure 7A,B and Supplementary Figure S2). Furthermore, the in vivo imaging system and H_2_DCFDA assay analyses showed that GCG pretreatment significantly inhibited the urban aerosol-induced increase in ROS in lung tissue, RAW264 cells, and inflammatory cells recovered from BALF (Figure 7C–E and Supplementary Figure S3). These results suggest that GCG is as effective as EGCG in preventing air-pollution-induced lung injury.

## 4. Discussion

Here, we investigated the preventive effect of EGCG, the main component of green tea, on acute lung injury caused by air pollutants. We found that EGCG prevented air-pollutant-induced increases in inflammatory cells (especially neutrophils) and inflammatory cytokines in the lungs. We also found that EGCG exerted its preventive effect by inhibiting the oxidative stress-dependent formation of NETs, as well as by acting as an antioxidant. In addition, GCG, a heat-epimerized form of EGCG, was found to exert a preventive effect against acute lung injury caused by air pollutants, similar to EGCG. These results suggest that EGCG and GCG prevent air-pollution-induced acute lung injury through their antioxidant effects and their inhibition of NET formation. The dosage of EGCG and GCG must be considered based on the results of this study if these compounds are to be practically applied as a preventive treatment for air-pollution-induced lung injury. Referring to a previous report [30], the human equivalent dose was calculated using the animal dose (100 mg/kg), animal weight (40 g = 0.04 kg), and human weight (60 kg), and we found that a person weighing 60 kg would need to ingest approximately 540 mg of EGCG or GCG per day. Although the catechin content varies among products, the calculated dose corresponds to the amount of catechins (catechins including EGCG and GCG) contained in approximately 700 mL of a beverage with a high catechin content (https://www.itoen-global.com/contents/en/oiocha/, accessed on 4 July 2022). Thus, we believe that daily consumption of green tea has the potential to reduce the health hazards caused by air pollution.

As described in the Introduction, no studies have analyzed the efficacy of EGCG against acute lung injury directly induced by air pollutants using a mouse model. However, several groups have reported the efficacy of catechins against air-pollution-induced health hazards using other types of models. Li, Y. et al. recently reported that PM_2.5_ administration exacerbated an ovalbumin-dependent asthma model via the high-mobility group box 1 pathway and that EGCG exerted an inhibitory effect on asthma exacerbation in that model [31]. In vitro analyses using BEAS-2B cells (airway epithelial cells) have also shown that the preadministration of catechins, such as EGCG, suppresses both cell injury and ROS production caused by airborne fine dust particles [32]. Other studies using human dermal fibroblasts and human umbilical vein endothelial cells have also reported that EGCG suppressed cell injury and ROS production caused by air pollutants by exerting antioxidant effects [33,34]. These results are in line with our findings and support our belief that the daily consumption of green tea may reduce the health hazards induced by air pollutants.

Previous studies have reported that air pollutants, such as PM_2.5_, can cause health hazards via ROS production [26,27,35], and our findings agree with those results. Thus, to further confirm that ROS production is the cause of lung injury caused by air pollutants, in our recent study, we established an in vivo imaging system to detect air-pollutant-dependent ROS production in the lungs [7]. As shown in Figure 4 and Figure 7, using this innovative system, we were able to demonstrate, for the first time, that EGCG and GCG directly inhibit air-pollutant-dependent ROS production in the lungs. Although the antioxidant effects of catechins have been reported in the lungs and other tissues, to the best of our knowledge, this is the first study to demonstrate the antioxidant effects of catechins using this innovative system. Stimuli such as ROS contribute to the exacerbation of various inflammatory diseases by overactivating NETs, which act as a defense system against bacterial and other infections [36,37]. The increased expression of NET markers has been reported in asthma, chronic obstructive pulmonary disease (COPD), and ARDS patients and has been correlated with disease severity [38,39,40]. For example, an analysis of sputum from COPD patients showed elevated NET marker expression in more than 90% of COPD patients with acute exacerbation, 45% of stable COPD patients, and 25% of smoker controls but in less than 5% of non-smokers [40]. Given this background of NET involvement in the exacerbation of various lung diseases, we recently used a mouse model to demonstrate the involvement of NETs in the development and exacerbation of air-pollution-dependent lung injury [7]. Although catechins were found to inhibit phorbol-myristate-acetate-dependent NET formation using an in vitro experimental system [41], no study has analyzed the effect of catechins on NETs formation using an air-pollution-induced lung injury model. Therefore, the results of this study are important with respect to the investigation of treatments to prevent air-pollution-dependent lung injury.

To establish EGCG or GCG treatment as a method for preventing the health problems caused by air pollution, several issues must still be resolved. In this study, we analyzed the effects of EGCG and GCG on lung injury directly caused by urban aerosol administration, but air pollutants are also reportedly involved in the exacerbation of various respiratory diseases. A 2016 meta-analysis found a significant association between short-term exposure and COPD exacerbation risk for all gaseous and particulate pollutants tested [42]. In addition, Ścibor, M. et al. found that particulate pollutants (PM_10_ and PM_2.5_) were associated with a significantly lower peak expiratory flow rate, an increased frequency of early asthma symptoms, and the use of fast-acting asthma inhalers [43]. Considering the findings of these reports, it is important to examine the effect of EGCG and GCG on the urban aerosol-induced exacerbation of various respiratory diseases. We found that a single intraperitoneal administration of EGCG or GCG was effective against acute lung injury caused by air pollutants. However, to apply this treatment for the prevention of health hazards caused by air pollution, it will be necessary to analyze the efficacy of EGCG and GCG when administered orally and when consumed over a long period of time, taking into consideration the case of ingestion through beverage consumption. Although few side effects are expected to occur from the long-term consumption of green tea [10], it will, nevertheless, be important to examine the safety of EGCG and GCG, in addition to their efficacy when ingested over a long period of time, to establish a safe and highly effective prevention method.

## 5. Conclusions

We found that EGCG exerts a preventive effect against urban aerosol-dependent acute lung injury and particularly reduced the neutrophil-mediated inflammatory response. We also found that the significant inhibition of urban aerosol-dependent ROS production is an important mechanism by which EGCG exerts its preventive effect. GCG, which is a thermal isomer of EGCG, was also found to exert a preventive effect against air-pollution-induced lung injury. Thus, we believe that foods and medications containing EGCG or GCG may be candidates to prevent the onset and progression of acute lung injury caused by air pollutants.

## Data Availability

The data that support the findings of our study are available from the corresponding author upon reasonable request.

## Supplementary Materials

The following supporting information can be downloaded at: https://www.mdpi.com/article/10.3390/biom12091196/s1. Figure S1: Primer sequences used in this study; Figure S2: Effect of EGCG on urban aerosol-dependent inflammatory responses; Figure S3: Effects of EGCG or GCG on urban aerosol-induced ROS production in inflammatory cells recovered in BALF; Figure S4: Effect of N-acetylcysteine on urban aerosol-induced ROS production; Figure S5: Effect of EGCG or GCG on urban aerosol-induced neutrophil extracellular trap formation; Figure S6: Original images for Western blotting analysis.
