# Supplementary material for: Preventive Effect of Epigallocatechin Gallate, the Main Component of Green Tea, on Acute Lung Injury Caused by Air Pollutants
Source: Biomolecules. 2022 Aug 29;12(9):1196. doi: 10.3390/biom12091196 (PMC9496336; doi:10.3390/biom12091196)
Supplement: Supplementary file 1 [file biomolecules-12-01196-s001.zip › biomolecules-1825259-supplementary.pdf]

Gene	Primer	Sequence (5' to 3')	Tm
TNF-alpha	Forward	CGTCAGCCGATTTGCTATCT	58.14
	Reverse	CGGACTCCGCAAAGTCTAAG	58.37
IL-1beta	Forward	GATCCCAAGCAATACCCAAA	55.67
	Reverse	GGGGAACTCTGCAGACTCAA	59.31
IL-6	Forward	CTGGAGTCACAGAAGGAGTGG	59.73
	Reverse	GGTTTGCCGAGTAGATCTCAA	57.75
Mip-2	Forward	ACCCTGCCAAGGGTTGACTTC	62.56
	Reverse	GGCACATCAGGTACGATCCAG	60.54
Kc	Forward	TGCACCCAAACCGAAGTCAT	60.18
	Reverse	TTGTCAGAAGCCAGCGTTCAC	61.41
Gapdh	Forward	AACTTTGGCATTGTGGAAGG	56.79
	Reverse	ACACATTGGGGGTAGGAACA	58.55

Supplementary Figure S1. Primer sequences used in this study.

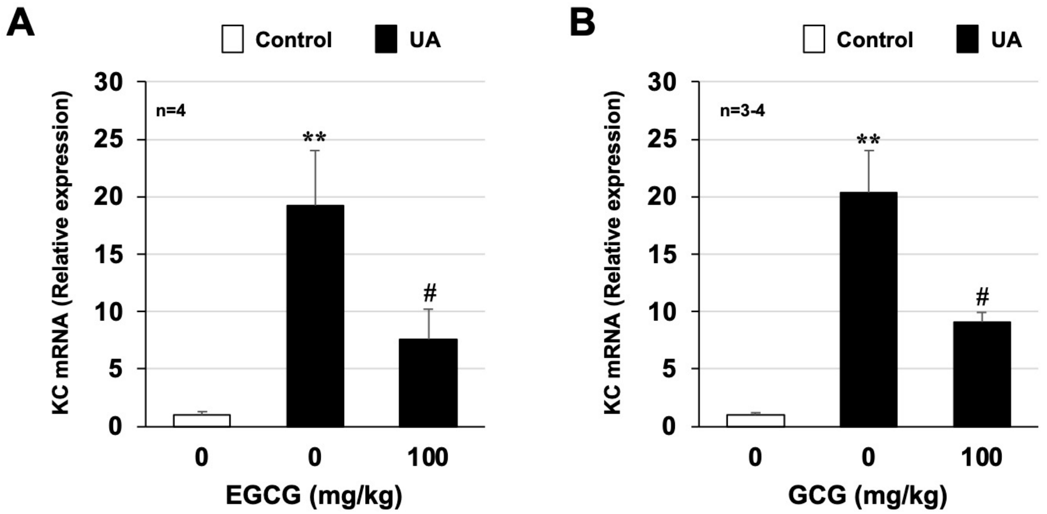

Supplementary Figure S2. Effect of EGCG on urban aerosol-dependent inflammatory responses. Male ICR mice were intraperitoneally administered EGCG (100 mg/kg in sterile saline), GCG (100 mg/kg in sterile saline), or sterile saline 1 h before intratracheal administration of urban aerosol particle suspension (1.0 mg/mouse) or sterile saline (Control). Total RNA was extracted from the lungs 24 h after urban aerosol particle suspension administration and subjected to real-time RT-PCR using a specific primer set for the keratinocytes-derived chemokine (*Kc*) gene. Values were normalized to *Gapdh* and are expressed relative to the Control. Values represent the mean ± SEM; #$p < 0.05$; **$p < 0.01$. (* vs Control; # vs UA).

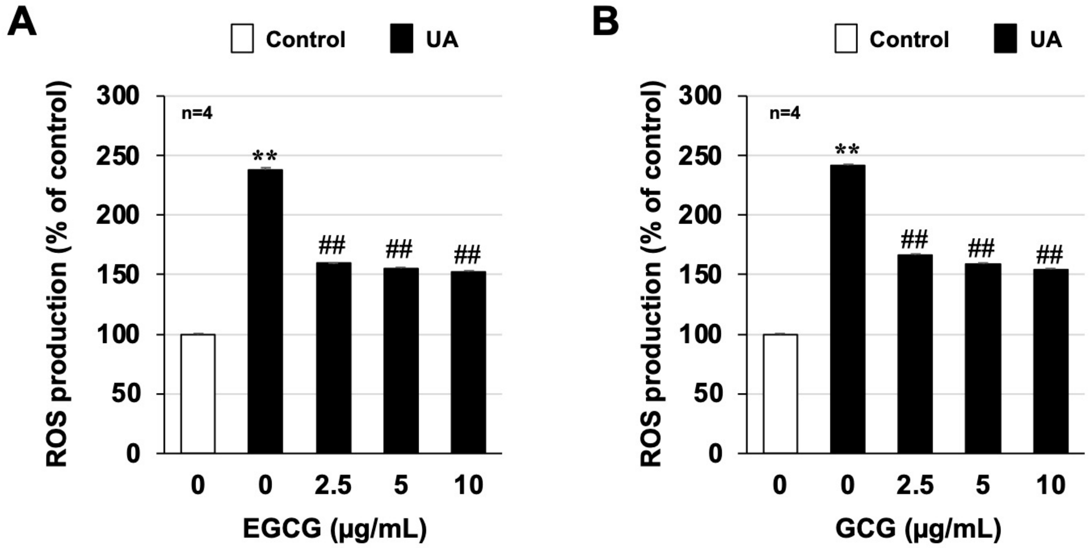

Supplementary Figure S3. Effects of EGCG or GCG on urban aerosol-induced ROS production in inflammatory cells recovered in BALF.
Male ICR mice were intratracheally administered urban aerosol particle suspension (1.0 mg/mouse) or sterile saline (Control). BALFs were prepared 24 h after the intratracheal administration. Inflammatory cells recovered from BALF (3.0 x 10^4 cells/well) were then seeded onto 96 well black plates and pre-cultured with H_2DCFDA (10 µM) for 60 minutes. The cells were treated with EGCG (2.5–10 µg/mL) or GCG (2.5–10 µg/mL) to the medium. After 24 h, the ROS levels were measured using a microplate reader. Values represent the mean ± SEM; ** or ## $p < 0.01$. (* vs Control; # vs UA).

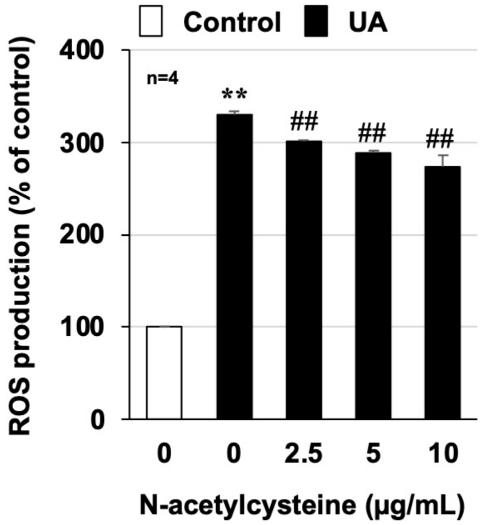

Supplementary Figure S4. Effect of N-acetylcysteine on urban aerosol-induced ROS production.
RAW264 cells were pre-cultured with H_2DCFDA (10 µM) for 60 min. Then, the cells were treated with N-acetylcysteine (2.5–10 µg/mL) prior to the addition of urban aerosols (30 µg/cm^2) to the medium. After 24 h, the ROS levels were measured using a microplate reader. Values represent the mean ± SEM; ** or ##$p < 0.01$. (* vs Control; # vs UA).

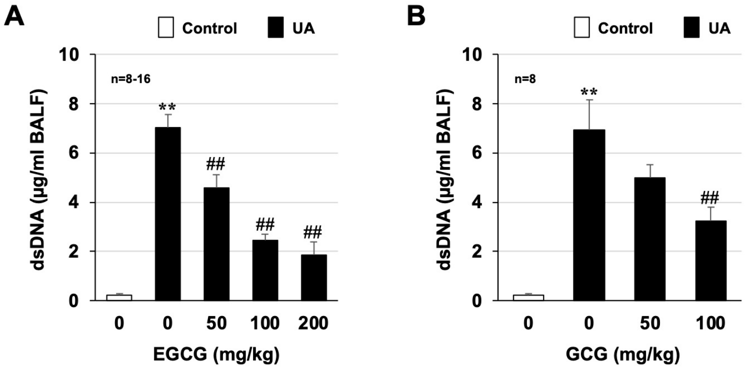

Supplementary Figure S5. Effect of EGCG or GCG on urban aerosol-induced neutrophil extracellular trap formation.

Male ICR mice were intraperitoneally administered EGCG (50–200 mg/kg in sterile saline), GCG (50-100 mg/kg in sterile saline), or sterile saline 1 h before intratracheal administration of urban aerosol particle suspension (1.0 mg/mouse) or sterile saline (Control). BALFs were prepared 24 h after the intratracheal administration. The amount of double-stranded DNA (dsDNA) present in the BALF was determined using the Quant-iT™ PicoGreen® dsDNA Assay Kits according to the manufacturer's protocol. Values are the mean ± SEM; ** or ##$p < 0.01$ (* vs Control; # vs UA).

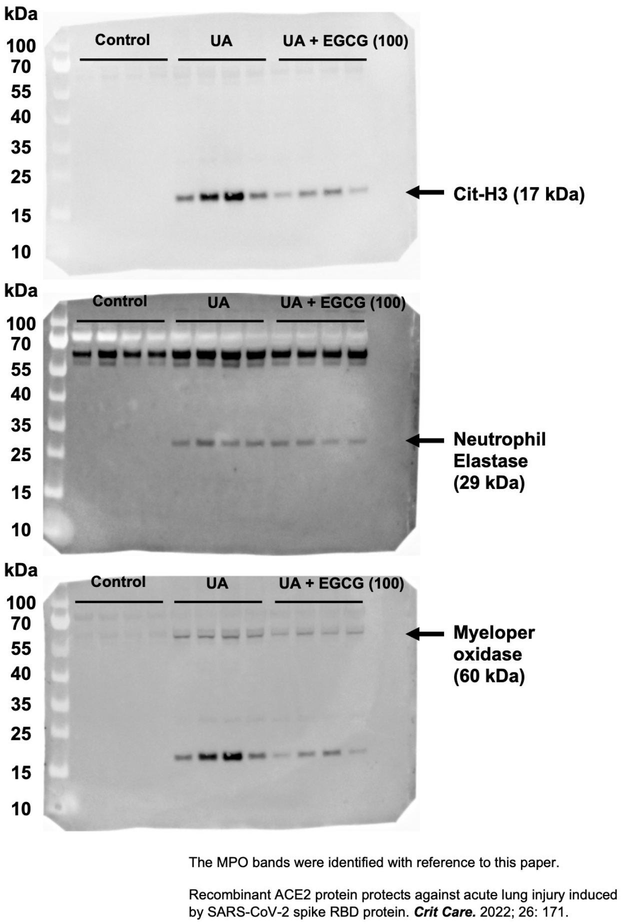

Supplementary Figure S6. Original images for Western blotting analysis.
